# The associations of cumulative adverse childhood experiences and irritability with mental disorders in detained male adolescent offenders

**DOI:** 10.1186/s13034-016-0122-7

**Published:** 2016-09-22

**Authors:** Hannes Bielas, Steffen Barra, Christine Skrivanek, Marcel Aebi, Hans-Christoph Steinhausen, Cornelia Bessler, Belinda Plattner

**Affiliations:** 1 Department of Child and Adolescent Psychosomatic Medicine and Psychotherapy, Clinic Fontane, Mittenwalde, Germany; 2 Department of Psychosomatics and Psychiatry, Child Protection Team, University Children’s Hospital Zurich, Zurich, Switzerland; 3 Department of Forensic Psychiatry, Centre for Child and Youth Forensic Service, University Hospital of Psychiatry, Neptunstrasse 60, Zurich, 8032 Switzerland; 4 University Clinics for Child and Adolescent Psychiatry, Paracelsus Medical University Salzburg, Salzburg, Austria; 5 Department of Child and Adolescent Psychiatry and Psychotherapy, University Hospital of Psychiatry, Zurich, Switzerland; 6 Division of Clinical Psychology with Children/Adolescents & Families/Couples, Department of Psychology, University of Zurich, Zurich, Switzerland; 7 Child and Adolescent Clinical Psychology, Institute of Psychology, University of Basel, Basel, Switzerland; 8 Child and Adolescent Mental Health Centre, Capital Region Psychiatry, Copenhagen, Denmark

**Keywords:** Childhood adversities, Emotion dysregulation, Juvenile offenders, Delinquent youth, Psychopathology

## Abstract

**Background:**

Adverse childhood experiences (ACEs) and psychiatric disorders are common in juvenile detainees. Emotional dysregulation resulting from cumulated ACEs may be characterized by symptoms of irritability. The present study examined whether the accumulation of ACEs, irritability, or both predicted mental disorders in incarcerated adolescents with and without controlling for one another and for socio-demographic factors.

**Methods:**

One hundred thirty male detained juvenile offenders (aged 13.8–19.5 years) were assessed by structured clinical interviews and a self-reporting scale for irritability. Univariate and multivariate regression models were used to examine the shared and distinct associations of ACEs and irritability with psychiatric diagnoses.

**Results:**

A total of 75 % of the participants reported more than one ACE. The ACE total score was positively related to self-reported irritability. The ACE total score predicted depressive disorders, suicidality, post-traumatic stress disorder (PTSD), and anxiety disorders. Irritability was positively related to depressive disorders, suicidality, disruptive behavior disorder (DBD), substance use disorder (SUD), and attention deficit hyperactivity disorder (ADHD). These associations remained significant in multivariate models.

**Conclusions:**

This study provides evidence for the predictive impact of self-reported ACEs and irritability with regard to adolescent psychiatric disorders in young male inmates. Both variables differed in their predictive power for PTSD, internalizing, and externalizing disorders indicating the need for specific therapeutic interventions. Taking a close look at their trauma history seems to be of special importance for juveniles suffering from PTSD and anxiety disorders. For delinquent adolescents with DBD, ADHD and SUD, the training of emotion regulation techniques appears most promising. Approaches focusing on both, ACEs and emotion-focused contents may be implemented in the treatment of depressive disorders and suicidality.

## Background

Adverse childhood experiences (ACEs) display a burden to children and adolescents worldwide with prevalence rates as high as 14–55 % for physical abuse, 11–47 % for emotional abuse, 6–22 % for sexual abuse, 7–19 % for physical neglect, and 15–40 % for emotional neglect [1]. However, ACEs are not restricted to these forms of maltreatment. The still ongoing ACE Study revealed high prevalence rates of up to 10 different types of childhood adversities in a large community sample [2, 3], additionally including domestic violence towards one’s mother, parental separation or divorce, living with someone in the houshold who is mentally ill, living with someone in the household who has substance abuse problems, and living with someone in the household who has been incarcerated.

The consideration of ACEs appears especially important in the context of juvenile delinquency. In their comprehensive study on more than 64,000 adolescent offenders, Baglivio and colleagues [4] found delinquent youths four times more likely to be burdened with four or more ACEs and 13 times less likely to have faced no ACE at all relative to the adult sample of the above-mentioned ACE study [2], and ACEs predicted an early onset and chronicity of offending [5, 6].

ACEs have been shown to play a crucial role in the development of mental health problems such as post-traumatic stress disorder (PTSD), anxiety, depression, and suicidal behaviors [7–10]. In addition, ACEs have been linked to externalizing problems such as antisocial behavior, interpersonal violence, delinquency, impulsivity, and ADHD [11–16], as well as substance abuse [17].

Chronic irritability is one of the core symptoms of emotion dysregulation in children and adolescents and a risk marker for the development of psychiatric disorders in later adolescence and adulthood [18, 19]. A child’s inability to cope with intense negative feelings and to regulate emotion has recently been defined as severe mood dysregulation disorder (DMDD) in DSM-5. A recent theory addressed the role of emotion regulation in the associations of early threatening and neglectful experiences with various psychiatric disorders [20]. According to this theory, ACEs relate to biased threat perceptions, which go along with enhanced reactivity of the autonomic nervous system as well as elevated neural responsivity to negative information. In other words, the early exposure to ACEs may impair children’s abilities to regulate their emotions manifesting in chronic, non-episodic irritability which was found to predict later affective and behavioral disorders. In line with this theory, Heleniak and colleagues [21] found in youth of a community sample that emotional dysregulation mediated the relations between maltreatment and psychopathology. In juvenile detainees, irritability was associated, amongst others, with antisocial, borderline, and narcissistic personality disorders [22], and was found to predict violent criminal re-offenses after release from detention [23]. By taking into account these recent findings, the present study addresses the specific impact of cumulative ACEs while controlling for irritability symptoms. To know how irritability and cumulative ACEs are related to distinct psychiatric disorders in detained adolescents is important for clinical decision making. For example, trauma related interventions should be provided specifically for detained youth with psychiatric disorders that arise from cumulative ACEs whereas emotion focused therapy or medication is indicated in youth with chronic irritability.

To the best of our knowledge, no study has yet investigated the effects of ACEs on psychiatric disorders in delinquent youth while considering the role of emotion dysregulation in terms of persistent irritability. The present study examined whether and how a cumulative score of ACEs and irritability predicted different psychiatric disorders in detained adolescents. Taking into account the high rates of both psychiatric morbidity and ACEs in detained juveniles [24–26], the present study (a) included various mental disorders, and (b) considered a variety of ACEs that have been examined in previous research. ACEs tend to occur in multiple forms [23, 27] and may have a cumulative effect on negative outcomes in terms of a dose–response relationship [2]. Furthermore, age at time of incarceration, foreign nationality, and low socio-economic status (SES) were included as common covariates of juvenile delinquency. Based on the above-mentioned literature review, we expected to obtain high rates of both ACEs and psychiatric disorders in the present adolescent detention sample. We also assumed that the cumulative scores of ACEs and irritability would positively predict internalizing and externalizing problems, and we hypothesized that ACE- and irritability-scores would be positively correlated.

## Methods

### Participants and procedure

The present study was conducted at the Zurich Juvenile Detention Centre, the only prison for male juvenile offenders in the Canton of Zurich (Switzerland). All juveniles consecutively admitted to this correctional facility between September 2010 and November 2012 were eligible for the present study. Exclusion criteria were (a) insufficient command of the German language; (b) significant medical conditions (e.g., acute state of human immunodeficiency virus, hepatitis, or other infectious diseases) and/or neurological disorders (e.g., epilepsy); and (c) intellectual disability or current psychotic symptoms (assessed by clinical impression). Data were assessed by four child and adolescent psychiatrists with special forensic training and one clinical forensic psychologist from the Department of Child and Adolescent Psychiatry, Zurich. The juveniles were invited for participation in the study within 5 days of admission.

Out of a total of 226 male juveniles, 31 (13.7 %) were excluded because of insufficient command of the German language, nine (3.9 %) were excluded because of intellectual disability/psychotic symptoms, and six (2.6 %) were excluded due to their release from detention prior to assessment. Four (1.8 %) juveniles refused to participate in the present study. Furthermore, 46 (20.3 %) adolescents were excluded because of missing or incomplete data. The age of the final sample consisting of 130 male adolescents ranged between 13.8 and 19.5 years (*M* = 16.84 years, *SD* = 1.15 years). Detention was due to the following self-reported main crimes: violent crimes (e.g., manslaughter, sexual coercion; *n* = 67, 51.5 %), property crimes (e.g., theft, defraud; *n* = 16; 12.3 %), drug related crimes (*n* = 1; 0.8 %), and other crimes (e.g., violation of current sanction; *n* = 46; 35.4 %).

### Measures

#### Adverse childhood experiences (ACEs)

ACEs were retrospectively assessed using the Multidimensional Clinical Screening Inventory for delinquent juveniles [MCSI; 24]. This semi-structured interview explores an adolescent’s psychosocial background combining forensic information and clinical history. The MCSI had been developed in discussion with leading juvenile delinquency experts and the instrument had been successfully implemented in previous research based on incarcerated youth samples [24, 28].

The MCSI includes the assessment of school and work history; behavioral problems at school; history of psychiatric disorders; previous psychiatric, psychological, and psychotherapeutic treatment; somatic history; psychiatric and neurological family history; marital status of the parents; placement in foster care institutions; and trauma. Out of all MCSI variables, only those adverse life events were considered for analysis that matched the 10 ACEs defined in the milestone study of Felitti et al. [2, 3; see Table 1] in order to assure comparability across studies. In accordance with previous research [e.g., 3], affirmative responses were summed up to compute a cumulative ACE total score (range = 0–10).

**Table 1:** ACE and psychiatric disorders in male detained adolescents

	Frequencies
ACE	
Emotional abuse	48 (36.9 %)
Physical abuse	53 (40.0 %)
Sexual abuse	3 (2.3 %)
Emotional neglect	24 (18.5 %)
Physical neglect	26 (20.0 %)
Battered mother	44 (33.8 %)
Parental separation or divorce	61 (46.9 %)
Mental illness in household	67 (51.5 %)
Household substance abuse	32 (24.6 %)
Incarcerated household member	62 (47.7 %)
Psychiatric disorders and suicidality	
PTSD	17 (13.1 %)
Anxiety disorders (w/o PTSD)	34 (26.2 %)
Depressive disorders	34 (26.2 %)
Substance use disorders	86 (66.2 %)
ADHD	60 (46.2 %)
Disruptive behavior disorders	104 (80.0 %)
Other disorders	3 (2.3 %)
Suicidality (moderate or severe)	34 (26.2 %)

*ACE* adverse childhood experiences, *PTSD* post traumatic stress disorder, *ADHD* attention deficit hyperactivity disorder

#### Psychopathology

Current psychiatric disorders were assessed using the structured Mini Neuropsychiatric Interview for Children and Adolescents [MINI-KID version 6.0; 29], which considers the diagnostic criteria of the Diagnostic and Statistical Manual of Mental Disorders, Fourth Edition (DSM-IV) and of the International Classification of Diseases (ICD-10). The present study included the following diagnoses: post-traumatic stress disorder (PTSD), depressive disorders (major depressive episode and/or dysthymia), substance use disorder (SUD), and disruptive behavior disorder (DBD; oppositional defiant disorder and/or conduct disorder), as well as ADHD and anxiety disorders (panic disorder, agoraphobia, separation anxiety disorder, social phobia, specific phobia, obsessive compulsive disorder). The MINI-KID has been proven to assess psychiatric disorders validly and reliably [23, 29]. Furthermore, the MINI-KID reliably reports current and lifetime suicidality (inter-rater and re-test reliabilities: AUC = 0.89–0.99, κ = 0.81–0.96) on three levels [low, moderate and severe; 29]. In the present study, suicidality was coded as present if rated as moderate or severe.

#### Covariates

Irritability was assessed using the German version of the Caprara Irritability Scale [CIS; 30, 31]. Statements of the 20 respective items were answered on 6-point Likert scales (1 = *not true*—6 = *exactly true*) and a cumulated irritability score was built (range 20–120). Reliability and validity of this self-report questionnaire has been demonstrated by the authors [30]. Internal consistency in the present sample was nearly excellent (Cronbach’s α = .88). Items of the CIS relate to affective and behavioral aspects of irritability as a result of the inability to control negative feelings (e.g., “It takes very little for things to bug me”, “I often feel like a powder keg ready to explode”, and “When I am tired I easily lose control”). General demographic data included age at time of incarceration, foreign (non-Swiss) nationality, and low SES. The latter was coded using the professional occupations of the adolescents’ mothers and fathers according to the International Standard Classification of Occupations (ISCO-08) guidelines [32]. Categories range from management positions (1) to unskilled workers (9); unemployment was coded as 10. Low SES was coded present when both caregivers had ISCO-scores of 9 and/or 10, or when one caregiver had a score of 9 or 10 while occupational information about the other was missing.

### Data analysis

Statistical analyses were conducted by use of SPSS 23. Descriptive methods were used to present the distributions of ACEs, the irritability score, psychiatric diagnoses, and demographic characteristics. Pearson’s correlation coefficients were calculated to quantify the relations between variables. Following Cohen’s [33] suggestions, effects were considered weak with coefficients smaller than .30, moderate with coefficients between .30 and .50, and strong with coefficients of at least .50.

Binary logistic regression analyses were used to examine the predictive effects of the ACE total score, the irritability score, the interaction of the ACE total score and the irritability score, age at time of incarceration, foreign nationality, and low SES on psychiatric diagnoses. In addition to unadjusted regression models, adjusted models were performed for each factor controlling for all other variables. Because the focus of the present study was on the presence of specific psychiatric diagnoses in detained adolescent offenders, we did not control for other co-occuring psychiatric disorders. For regression analyses, numeric scale scores (ACE total score, irritability score, and age at time of incarceration) were z-transformed in order to facilitate interpretation. Multivariate analyses were performed to buffer against type 1 error. Multicollinearity was checked by inspecting the correlation matrix of all variables as well as the variance inflation factor (VIF) and the tolerance values. No multicollinearity issues were assumed when intercorrelations were low to medium, VIF values below 10, and tolerance values below .10 [34]. Because of the exploratory character of the present study we also included statistical trends in our findings.

## Results

### Descriptive findings

#### Sample characteristics

The participating 130 incarcerated males were equally likely of Swiss (*n* = 72, 55 %) or foreign nationality (*n* = 58, 45 %; χ^2^(1) = 1.51, *p* = .22). A quarter of the juveniles (*n* = 32) were of low SES. A marginally significant association was found between foreign nationality and low SES (χ^2^(1) = 3.573, *p* = .06). In comparison to the remaining participants, the 96 drop-outs were older (*M* = 17.14 vs.16.83 years, *t*(224) = 2.03, *p* = .04) and more likely of foreign nationality (*n* = 60, 62.5 % vs. *n* = 58; 44.6 %, χ^2^(1) = 7.08, *p* < .001). There were no significant differences between participants and drop-outs as for low SES (*n* = 13, 13.5 % vs. *n* = 22, 16.9 %; χ^2^(1) = 0.482, *p* = .49). The proportions of violent and other crimes were significantly higher for included juveniles (adjusted residuals: 8.9 and 4.6, respectively), whereas excluded juveniles showed more property and drug related crimes (adjusted residuals: 7.6 and 5.9, respectively; χ^2^(3) = 22.03, *p* < .001).

#### ACEs

Of all participants, 91.5 % reported at least one ACE, while about 75 % of the participants reported more than one ACE. The frequencies of affirmed ACEs are shown in Table 1. Mental illness in the household appeared to be the most prevalent ACE, followed by an incarcerated household member, and parental separation/divorce. The least common ACEs included sexual abuse and emotional neglect. The mean ACE total score was 3.22 (*SD* = 2.15) ranging from 0 to 9.

#### Psychopathology

The frequencies of adolescent psychiatric disorders according to the MINI-KID are also shown in Table 1. The majority of the incarcerated adolescents fulfilled the diagnostic criteria for DBD, followed by SUD. Approximately a quarter of the participants suffered from depressive disorders and suicidality. PTSD was diagnosed in 13 % of the sample. Comorbities were frequent in the present sample as 79.2 % (*n* = 103) of the juveniles showed two or more co-existing diagnoses (*M* = 2.80, *SD* = 1.73, range = 0–7).

#### Irritability

The mean self-rated irritability score was 61.78 (*SD* = 17.34) ranging from 24 to 106. The irritability score was significantly and positively correlated with the ACE total score (*r* = 0.19, *p* = .03).

### Results of the prediction analyses

Multicollinearity was not an issue in the present analyses since intercorrelations were low to moderate (Table 2), and the VIF and tolerance values did not exceed respective cut-offs (ranges: 1.02–1.08, and .93–.98, respectively). Tables 3, 4 and 5 display the results of the binary regression analyses. The ACE total score was a consistent predictor of PTSD, anxiety disorders, depressive disorders, and suicidality in both the unadjusted and adjusted models. The ACE total score was not predictive of DBD and ADHD, and only by trend a significant predictor for SUD in the unadjusted model. The irritability score consistently predicted depressive disorders, suicidality, DBD, ADHD, and SUD. For anxiety disorders, its predictive value was significant only by tendency in the unadjusted model and not significant in the adjusted model. The irritability score did not predict PTSD. The interaction term of the ACE total score and the irritability score did not reach statistical significance, neither in univariate nor in multivariate models.

**Table 2:** Correlation matrix for psychiatric diagnoses, ACEs, and covariates in detained adolescent offenders

	PTSD	Anxiety disorders	Depressive disorders	Suicidality	SUD	DBD	ADHD	ACE total score^a^	Irritability^a^	Age^a^	Foreign nationality	Low SES
PTSD	1	.029	.392^***^	.285^**^	.085	.023	.053	.279^**^	.150^+^	−.080	.203^*^	.031
Anxiety disorders		1	.323^***^	.354^***^	.167^+^	.123	.221^*^	.224^*^	.156^+^	.040	−.041	.055
Depressive disorders			1	.480^***^	.315^***^	.123	.186^*^	.338^***^	.222^*^	.075	.100	.051
Suicidality				1	.188^*^	.129	.134	.263^**^	.202^*^	.076	.002	.051
SUD					1	.333^***^	.173^*^	.173^*^	.237^**^	.243^**^	−.012	−.063
DBD						1	.347^***^	.097	.319^***^	−.004	.023	−.045
ADHD							1	.120	.244^**^	.016	.131	−.094
ACE total score^a^								1	.192^*^	−.025	−.014	−.195^*^
Irritability^a^									1	−.099	−.101	−.166^+^
Age^a^										1	.075	−.051
Foreign Nationality											1	.087
Low SES												1

*PTSD* post traumatic stress disorder, *SUD* substance use disorder, *DBD* disruptive behavior disorder, *ADHD* attention deficit hyperactivity disorder, *ACE* adverse childhood experience, *SES* socio-economic status

Significance (two sided), ^+^ *p* < .10, * p < .05, ** p < .01, *** *p* < .001

^a^ z-Transformed

**Table 3:** Predicting the presence of PTSD and other anxiety disorder by ACEs and covariates in detained adolescent offenders

Variables	PTSD		Other anxiety disorder	
	Unadjusted model	Adjusted model^a^	Unadjusted model	Adjusted model^a^
	OR (95 % CI)	OR (95 % CI)	OR (95 % CI)	OR (95 % CI)
ACE total score^b^	2.28 (1.33–3.91)**	2.39 (1.29–4.46)**	1.67 (1.12–2.49)**	1.68 (1.10–2.57)*
Irritability score^b^	1.59 (0.93–2.73)	1.63 (1.29–4.46)	1.44 (0.96–2.17)^+^	1.44 (0.92–2.26)
ACE total score^b^ * Irritabiliy score^b^	1.14 (0.72–1.80)	0.83 (0.46–1.49)	1.03 (0.71–1.48)	1.04 (0.68–1.58)
Age at incarceration^b^	0.79 (0.47–1.32)	0.80 (0.46–1.40)	1.10 (0.74–1.63)	1.21 (0.79–1.85)
Foreign nationality	3.50 (1.15–10.59)*	4.32 (1.30–14.31)*	0.83 (0.38–1.83)	0.76 (0.33–1.78)
low SES	0.61 (0.16–2.29)	0.50 (0.12–2.11)	0.72 (0.30–1.74)	1.60 (0.63–4.09)

*PTSD* posttraumatic stress disorder, *ACE* adverse childhood experience, *SES* socio-economic status, *CI* confidence interval, *OR* odds ratios

^a^Adjusted for demographics, ACE total score, irritability score, and interaction term ACE total score * irritability score

^b^ z-Transformed

Significance (two sided), ^+^ *p* < .10, * *p* < .05, ** *p* < .01, *** *p* < .001

**Table 4:** Predicting the presence of depressive disorders and suicidality by ACEs and covariates in detained adolescent offenders

Variables	Depressive disorders		Suicidality	
	Unadjusted model	Adjusted model^a^	Unadjusted model	Adjusted model^a^
	OR (95 % CI)	OR (95 % CI)	OR (95 % CI)	OR (95 % CI)
ACE total score^b^	2.23 (1.45–3.45)***	2.33 (1.44–3.78)**	1.89 (1.23–2.91)***	1.95 (1.21–3.14)**
Irritability score^b^	1.72 (1.12–2.63)**	1.82 (1.10–2.96)*	1.67 (1.07–2.61)*	1.78(1.07–2.99)*
ACE total score^b^ * Irritabiliy score^b^	1.14 (0.79–1.63)	1.04 (0.64–1.69)	1.05 (0.71–1.54)	0.98 (0.62–1.57)
Age at incarceration^b^	1.19 (0.80–1.77)	1.36 (0.86–2.16)	1.20 (0.80–1.83)	1.40 (0.87–2.25)
Foreign nationality	1.58 (0.72–3.46)	1.76 (0.72–-4.29)	1.01 (0.44–2.32)	0.95 (0.38–2.34)
low SES	1.10 (0.44–2.75)	0.95 (0.35–2.59)	0.66 (0.27–1.66)	1.83 (0.67–4.97)

*ACE* adverse childhood experience, *SES* socio-economic status, *CI* confidence interval, *OR* odds ratios

^a^Adjusted for demographics, ACE total score, and irritability score, and interaction term ACE total score * irritability score

^b^z-Transformed

Significance (two sided), * p < .05, ** p < .01, *** p < .001

**Table 5:** Predicting the presence of disruptive behavior disorder, ADHD, and substance use disorder by ACEs and covariates in detained adolescent offenders

Variables	Disruptive behavior disorder		ADHD		Substance use disorder	
	Unadjusted model	Adjusted model^a^	Unadjusted model	Adjusted model^a^	Unadjusted model	Adjusted model^a^
	OR (95 % CI)	OR (95 % CI)	OR (95 % CI)	OR (95 % CI)	OR (95 % CI)	OR (95 % CI)
ACE total score^b^	1.29 (0.82–2.03)	1.07 (0.63–1.83)	1.28 (0.90–1.81)	1.15 (0.79–1.69)	1.47 (1.00–2.17)^+^	1.42 (0.92–2.19)
Irritability score^b^	2.40 (1.46–3.95)***	2.39 (1.41–4.05)**	1.68 (1.16–2.45)**	1.70 (1.15–2.52)**	1.69 (1.15–2.51)**	1.76 (1.15–2.60)**
ACE total score^b^ * Irritabiliy score^b^	0.86 (0.58–1.27)	0.90 (0.53–1.54)	1.09 (0.78–1.51)	1.06 (0.72–1.56)	0.96 (0.68–1.35)	1.02 (0.66–1.58)
Age at incarceration^b^	0.99 (0.64–1.53)	1.03 (0.64–1.64)	1.03 (0.73–1.46)	1.06 (0.73–1.53)	1.71 (1.16–2.54)**	1.92 (1.26–2.92)**
Foreign nationality	1.12 (0.47–2.68)	1.52 (0.58–4.00)	1.70 (0.85–3.42)	1.98 (0.94–4.19)	0.95 (0.46–1.97)	0.93 (0.41–2.10)
low SES	1.15 (0.43–3.05)	0.86 (0.30–2.52)	0.94 (0.42–2.10)	1.00 (0.43–2.33)	1.02 (0.44–2.36)	1.06 (0.42–2.66)

*ADHD* attention deficit hyperactivity disorder, *ACE* adverse childhood experience, *SES* socio-economic status, *CI* confidence interval, *OR* odds ratios

^a^Adjusted for demographics, ACE total score, and irritability score, and interaction term ACE total score * irritability score

^b^z-Transformed

Significance (two sided), ^+^ *p* < .10, * p < .05, ** p < .01, *** p < .001

Age at time of incarceration was only predictive of SUD in both the unadjusted and the adjusted model. Foreign nationality consistently predicted PTSD. Low SES did not predict any psychiatric disorder.

## Discussion

To the best of our knowledge, the present study is the first examination of the predictive impact of cumulative ACEs on various psychiatric disorders in juvenile detainees while accounting for the effects of irritability and demographic covariates. As expected and in line with studies on detained juveniles in other countries [24, 25, 35], high rates of ACEs and psychiatric disorders were found in the present Swiss sample. The results underscore that most delinquent juveniles are highly burdened with ACEs and psychopathology. However, considering the high prevalence of multiple ACEs in the current sample (more than 75 %), it is rather surprising that only a relatively low percentage of juveniles fulfilled the diagnostic criteria for PTSD (13 %). Interestingly, recent studies found similar prevalence rates for PTSD (10–20 %) in delinquent youth [25, 36–38]. Given the biological impact of traumatic stress, it may be assumed that even if only some adolescents develop the classical symptoms and fulfill diagnostic criteria of PTSD, other mood and anxiety symptoms may be present as well [39]. However, the latter were not detected in the present study by the MINI-KID that goes only for full-blown diagnoses without considering subthreshold manifestations.

The ACE total score predicted PTSD, anxiety disorders, depressive disorders, and suicidality. Odds ratios indicated that the probabilities of these diagnoses were approximately doubled when the ACE total score increased by one standard deviation. These findings remained significant even when taking symptoms of irritability into account. Our findings suggest that there is a direct link between the exposure to multiple adversities in childhood and adolescence and the development of internalizing psychiatric disorders. These results support previous findings on dose–response relationships between ACEs and several dysfunctional outcomes [e.g., 2]. Given the impact of multiple negative experiences on psychopathology, juveniles suffering from mood and anxiety disorders may benefit from trauma-focused treatment approaches, such as narrative exposure therapy for forensic offender rehabilitation [FORNET; 40].

In contrast, the cumulative ACE score was not associated with DBD and ADHD, and was related to SUD only by tendency. This finding was somewhat unexpected given the broad reaching effects of cumulative ACEs (Duke 2010) and the high rates of ACEs in detained youth in previous studies [23, 41]. However, because of the high rates of both ACEs and externalizing disorders in the present sample we cannot exclude that ceiling-effects may have influenced our results.

In line with previous research [e.g., 21], irritability was found to play a prominent role in the prediction of externalizing as well as internalizing disorders in detained male adolescents. The finding of irritability as the sole predictor of ADHD is comprehensible, given the neurobiological foundation of the disorder and the predictive impact of infant temperament and emotional dysregulation on ADHD [42]. The association of irritability with SUD may be due to to the function of substance use as self-medication in order to treat the effects of irritability [43]. These results suggest the need for rather specific therapeutic treatment options with a focus on emotional dysregulation, e.g., dialectical behavior therapy [44] in addition to social work interventions which are still indicated in the vast population of delinquent juveniles with diagnoses of DBD, ADHD, and SUD.

Besides its role in the manifestation of disruptive behavior disorders, irritability is also a core symptom of affective disorders and is a major risk factor for suicidality [e.g., 45, 46]. Suicide prevention is a major challenge in the prison system. Considering the fact that only depressed juveniles generally mobilize suicide prevention measures, irritability might be misinterpreted as an endangerment to others only, but not as a risk factor for self-harm. Behavioral psychotherapy focusing on irritability may be expected to yield positive results in juvenile detainees. Furthermore, psychopharmacological treatment might be helpful for extremely irritable individuals [47].

The present study indicates that ACEs and irritability appear to contribute to the development of psychiatric disorders and behavior problems by both shared and specific effects depending on the type of disorder. Our results revealed a weak but significant correlation between irritability and cumulative ACE scores indicating that the effects of ACEs and irritability on psychopathology may be dependent on each other at least to some extent. However, the results of the regression analyses underscore that both irritability and cumulative ACEs are distinctively contributing to the high prevalence of psychopathology in detained youths. The findings of the present study partly support recent theories on pathways that explain the relations between ACEs and psychopathology through collateral effects of emotion regulation [e.g., 20]. However, taking into account that irritability predicted externalizing problems even though ACEs did not (neither in the unadjusted nor the adjusted models), some assumptions on the mediating role of irritability in the associations of ACEs and psychopathology are challenged by the present findings.

Among the various covariates, foreign nationality was associated with an almost four-fold risk of having PTSD. Theses results are in line with recent data which have shown a link between migration and psychiatric disorders in a Swiss sample [48]. The mechanisms behind this phenomenon should be studied in further research given the ongoing discussion on the integration of foreign youth. The chances for having SUD increased with older age reflecting the vicious circle between crime and substance abuse behavior [49].

The present findings must not be interpreted without the consideration of various limitations. First, the sample consisted of a single juvenile correctional facility in Switzerland, which limits generalization to other correctional facilities in other countries. Secondly, included participants were not fully representative of the entire clientele by showing lower proportions of foreign nationality, younger age, and different crime distributions compared to excluded juveniles. Additionally, the results might not be applicable to individuals who have committed less serious or minor criminal acts that had not led to incarceration. Furthermore, it may not be fully excluded that self-reports caused some bias in the present data due to fallibility of memory, social desirability, and avoidance of reporting family dysfunction. The dichotomous assessment of life-time ACEs did not respect their severity and/or chronicity. Although the psychometric properties of the English version of the MINI-KID had been shown to be good, we are not aware of any psychometric evaluations of the German version. Our study included juvenile detainees up to the age of 19. These juveniles were also assessed using the kids version of the MINI because our clinicians considered the wording and questioning appropriate and were able to individually adapt language in an age appropriate manner. The MCSI was reported in two prior studies on an Austrian sample [24, 28]. Still, psychometric properties are not yet available. Finally, the present study does not allow any causal inferences due to its cross-sectional design; factors not controlled for in the present analyses may also exert essential influences on the outcome variables. In particular, symtoms of ADHD and DBD may overlap to a certain extent with symptoms of irritability.

In conclusion, several clinical implications may be derived from the present findings. Delinquent youths with different psychiatric disorders may need specific interventions tailored to their needs. Assessment and treatment of PTSD and anxiety disorders in adolescent detainees should refer to the trauma history of the adolescent and should consider a broad range of different ACEs. Approaches focusing primarily on the treatment of emotion regulation may be most appropriate for juveniles with DBD, ADHD, and SUD. Treatment of depressive disorders and suicidality may require the implementation of both trauma and emotion-focused contents.

## Abbreviations

ACE: adverse childhood experience
ADHD: attention deficit hyperactivity disorder
CI: confidence interval
CIS: Caprara Irritability Scale
DBD: disruptive behavior disorder
DSM-IV: Diagnostic and Statistical Manual of Mental Disorders, fourth edition
FORNET: narrative exposure therapy for forensic offender rehabilitation
ICD-10: International Classification of Diseases, tenth edition
ISCO-08: International Standard Classification of Occupations guidelines
MCSI: multidimensional clinical screening inventory for delinquent juveniles
MINI-KID: mini neuropsychiatric interview for children and adolescents
OR: odds ratios
PTSD: post-traumatic stress disorder
SES: socio-economic status
SUD: substance use disorder
